# Preoperative proton pump inhibitor therapy and anastomotic leak after esophagectomy–a new perspective

**DOI:** 10.1007/s00423-025-03727-3

**Published:** 2025-05-14

**Authors:** Lukas Pollmann, Jonas Linnemann, Nicola S. Pollmann, Claudius Jürgens, Maximilian Schmeding

**Affiliations:** 1 Department of General and Visceral Surgery, Klinikum Dortmund gGmbH, Dortmund, Witten/Herdecke University, Beurhausstraße 40, 44137 Dortmund, Germany; 2 Department of General-, Visceral and Transplant Surgery, University Hospital Jena, Am Klinikum 1, 07747 Jena, Germany

**Keywords:** Anastomotic leak, Esophagectomy, Proton pump inhibitor, Preoperative management

## Abstract

**Purpose:**

Proton pump inhibitors (PPIs) are indispensable in the treatment of gastro-esophageal reflux disease and peptic ulcers or for the prevention of stress ulcers after major abdominal surgery. However, long-term PPI therapy leads to several side effects such as delayed gastric emptying and distinct changes in mucosal histology. Therefore, this retrospective study aims to evaluate the impact of preoperative PPI therapy on the anastomotic leak rate after esophagectomy.

**Methods:**

A retrospective, single-center analysis was conducted for all patients treated with esophagectomy and gastric conduit reconstruction between January 2016 and November 2024. Preoperative treatment with PPIs, as well as patient comorbidities, histopathological findings and surgical techniques were noted. Subsequently, a group-wise comparison was carried out for the differences in anastomotic leak rate and postoperative complications in patients with and without preoperative PPI therapy. Finally, a multivariate logistic regression analysis was conducted for the occurrence of anastomotic leak.

**Results:**

A total of 229 patients were included in the study. The group-wise comparison revealed a significantly higher rate of anastomotic leaks and postoperative complications in patients with preoperative PPI therapy compared to those without. The multivariate logistic regression analysis indicated a 2.5-fold increased risk of anastomotic leaks in patients with preoperative PPI therapy compared to patients without.

**Conclusion:**

Preoperative PPI therapy may represent a modifiable risk factor for the development of anastomotic leaks after esophagectomy. Further prospective, interventional studies are necessary to verify the results.

**Trial registration:**

The study was retrospectively registered in the German clinical trial database (Application number DRKS00035536, Registration date 03.12.2024).

**Graphical abstract:**

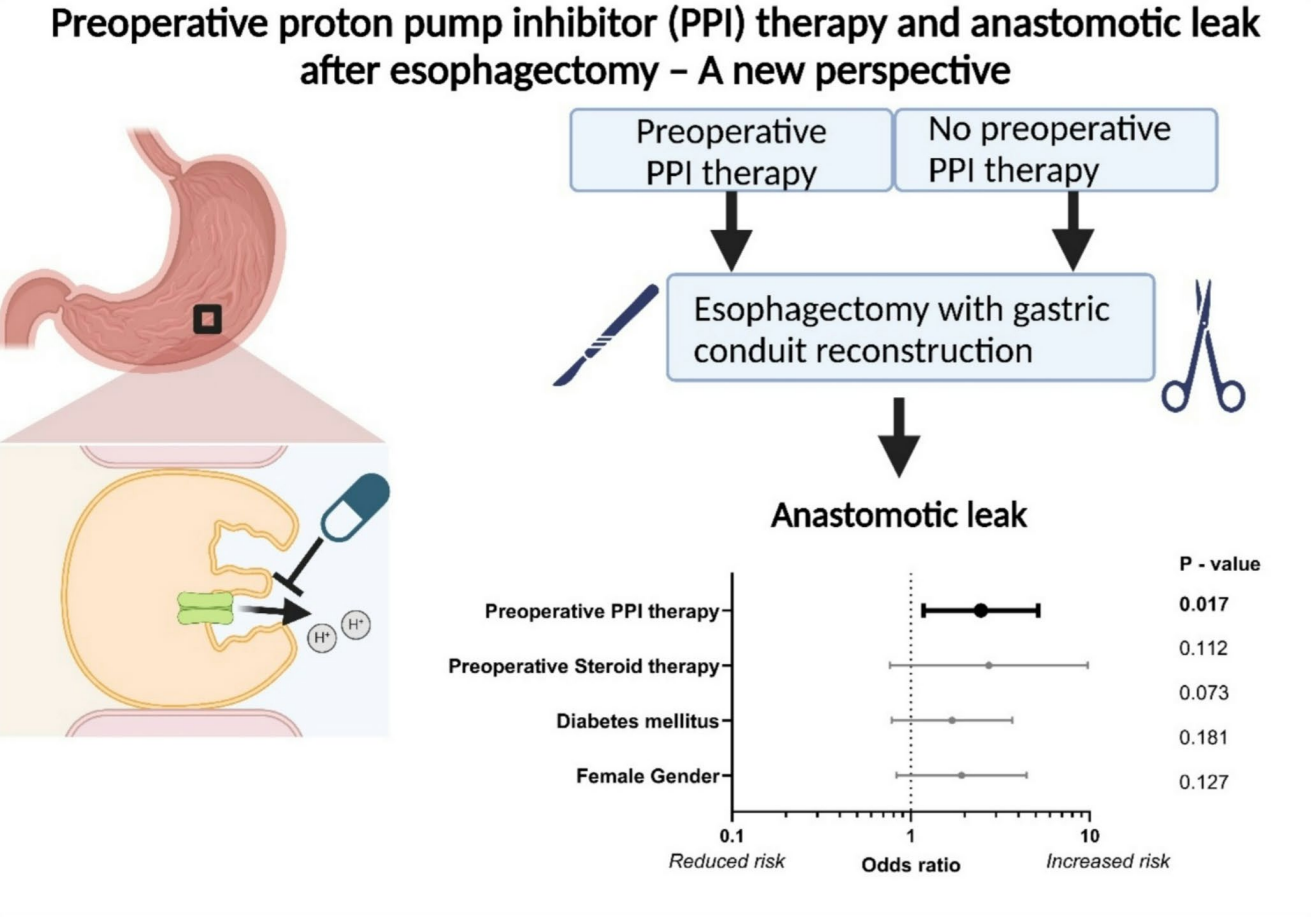

**Supplementary Information:**

The online version contains supplementary material available at 10.1007/s00423-025-03727-3.

## Introduction

Proton pump inhibitors (PPIs) represent an integral part of modern healthcare due to their various indications. As PPIs reduce acid production by inhibiting gastric parietal cells, they constitute the first-line therapy for treating the symptoms of gastro-esophageal reflux disease [1]. In addition to antibiotic therapy, PPI therapy constitutes the primary method of treating Helicobacter pylori infection and gastroduodenal ulcer disease [2]. Moreover, they prevent the metaplasia of esophageal mucosa into Barrett’s mucosa when exposed to gastric acid. Furthermore, perioperative PPI therapy is frequently used to prevent stress ulcer after major abdominal surgery [3]. After gastrointestinal anastomosis– for example as a part Roux-y-gastric bypass surgery [4] or pancreatoduodenectomy [5]– PPI therapy is the therapy of choice for the treatment and prevention of marginal ulcers.

The reconstruction after esophagectomy, according to Ivor-Lewis or McKeown, often includes formation of a gastric conduit. Although the gastric conduit is denervated from the vagal nerve during the procedure, it recovers gastral acidity with time [6]. Subsequently, anastomosis can be exposed to gastric acid reflux [7]. As a long-term complication of gastric acid reflux, anastomotic leak, or reduced blood supply an anastomotic stricture may occur [8]. In cases where gastric reflux is a contributing cause, PPIs have proven to be effective in reducing the symptoms of acid reflux and the rate of stenosis of the esophagogastric anastomosis [9].

However, as a local effect on gastric physiology, an increase in serum gastrin level has been noted during long-term PPI therapy due to impaired feedback mechanisms of the gut-brain axis [10, 11]. Furthermore, gastric emptying in healthy subjects under PPI therapy was delayed compared to healthy subjects without exposure to PPI therapy [12]. A histological examination of the stomach indicated that remodeling processes with an increase in Enterochromaffin-like cells occurred during long-term PPI therapy. Additionally, fundic gland polyps and hyperplastic polyps were described as the endoscopic findings of parietal cell protrusion and foveolar epithelial hyperplasia [13]. As delayed gastric emptying and changes in gastric, mucosal histology might impair the anastomotic healing [14, 15], the impact of preoperative PPI therapy on anastomotic leak rate and overall perioperative complications should be further investigated in the current study.

## Materials and methods

### Retrospective data acquisition and ethical statement

All patients with esophageal carcinoma, who were treated by esophagectomy and gastric conduit reconstruction at our clinic from January 2016 to November 2024, were identified. Patients with other causes for esophagectomy, such as esophageal perforation, were excluded. Furthermore, patients with colonic interposition for esophageal replacement were excluded. First, frequent, preoperative use of PPI was determined based on the medication plan and medical history form at the time of admission. Furthermore, self-reported PPI intake was noted. A daily PPI intake was required for the inclusion in the PPI group. No subgrouping for different PPIs, e.g. Pantoprazol, Omeprazol, Esomeprazol etc., was performed. Furthermore, no dose-dependent subgroup analysis was done. The usage of other gastric acid suppressants, like histamine type 2 receptor antagonists, was not analyzed in the current study. Baseline characteristics included age, body mass index (BMI) and gender. Additionally, patient comorbidities related to the development of anastomotic leak after esophagectomy [16, 17], e.g. arterial hypertension, chronic obstructive pulmonary disease (COPD), diabetes mellitus, heart disease, vascular disease, cardiac arrhythmia, chronic kidney disease and preoperative steroid therapy were identified. Moreover, any history of gastritis or peptic ulcers and gastro-esophageal reflux disease was noted. Finally, neoadjuvant treatment, the tumor- (T), node- (N) and metastases (M) status and residual tumor status (R-status), as well as the surgical technique and the type of anastomosis (e.g. circular stapled or suture anastomosis) were recorded. The study was approved by the institutional ethics committee (Ethics committee Witten/Herdecke; Application number: 292/2024, Date of approval: 05.11.2024). Furthermore, the study was registered in the German clinical trial database (Application number: DRKS00035536).

### Analysis of patient characteristics and outcome parameters

Afterwards, a group-wise comparison of the baseline characteristics in patients with preoperative PPI intake and patients without preoperative PPI intake was performed. Differences of categorical variables were analyzed using Fisher’s exact test. For the comparison of differences in patient age, the student t-test was used. The Mann–Whitney U test was used for the comparison of BMI between patients with and without preoperative PPI intake. For all tests of baseline characteristics, no correction for multiple testing was performed.

The primary outcome parameter was the occurrence of anastomotic leaks. An anastomotic leak is defined as a full-thickness gastrointestinal defect involving the esophagus, anastomosis, staple line, or gastric conduit, as stated by the Esophagectomy Complications Consensus Group [18]. The diagnosis was made clinically, radiologically, or endoscopically. The difference in the anastomotic leak rate of patients with and of those without preoperative PPI therapy was compared using the Fisher’s exact test. Furthermore, the comprehensive complication index (CCI) was estimated using the Clavien–Dindo-classification of postoperative complications. In addition, the length of hospital stay was accessed for all the patients discharged from hospital. The length of hospital stay was not calculated for patients, who were admitted to other departments or nursing facilities as well as for patients who died in hospital. As the length of hospital stay and the CCI were not normally distributed in both groups, a comparison of the differences was performed using the Mann–Whitney U test.

### Further statistical evaluation

After the group-wise comparison, a logistic regression analysis was performed with anastomotic leak rate as the dependent variable and the assumed risk factors for anastomotic leak, e.g. PPI intake, age, gender, BMI, arterial hypertension, chronic obstructive pulmonary disease (COPD), diabetes mellitus, heart disease, vascular disease, cardiac arrhythmia, chronic kidney disease and preoperative steroid therapy, as independent variables. All independent variables, with the exception of preoperative PPI intake, have been discussed as risk factors for the development of anastomotic leak in previous studies [16, 17]. A backward selection method was chosen. An independent variable was entered if p-value < 0.05 and the variable was removed if p-value > 0.2.

Furthermore, multiple linear regression was carried out for the mentioned independent variables and the development of postoperative complications. Again, a backward selection was performed. Variables were included in the model if the p-Value < 0.05 and removed if p-value > 0.1.

**Fig. 1:**
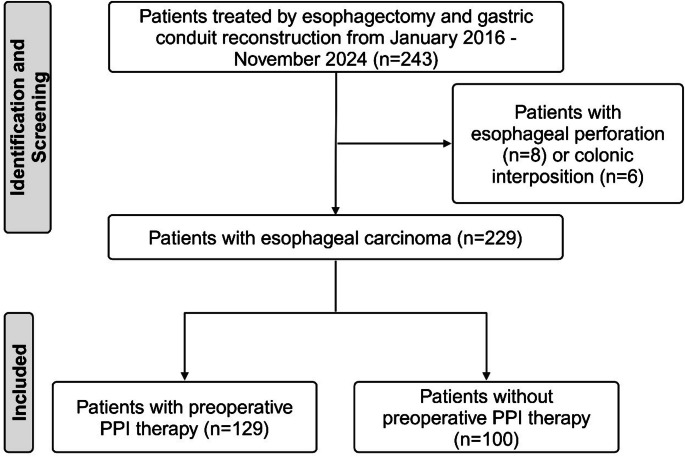
Flow diagram for patient identification, screening and inclusion in the current study

To assess the severity of anastomotic leaks, the proportions of different interventions to treat anastomotic leaks were recorded. In addition, the severity of the anastomotic leaks was classified according to the Clavien-Dindo classification of postoperative complications. All statistical analyses were performed using the Medcalc software (Medcalc Software Ltd.); data was visualized using the Graphpad software (GraphPad Software, Inc) and Fiji open-source software [19]. Biorender (Biorender, Toronto, Canada) was utilized to create the graphical abstract and Fig. 1.

## Results

### Patient characteristics

After the screening, 229 patients were included in the study, see Fig. 1. The mean patients age was 65 ± 9.6 years (mean ± standard deviation), mean BMI was 26 ± 5.5 kg/m² and only 38 patients were female. The main patient comorbidities were arterial hypertension, diabetes mellitus and the presence of any heart disease. Consequently, the most common American Society of Anesthesiologists (ASA) score in the study cohort was III. The surgical technique varied from open Ivor Lewis esophagectomy to hybrid techniques using laparoscopy and thoracotomy for Ivor Lewis esophagectomy to full robotic Ivor Lewis esophagectomy. All gastroesophageal anastomosis performed during Ivor Lewis esophagectomy were intrathoracic (*n* = 212). Furthermore, 17 patients underwent McKeown esophagectomy with cervical gastroesophageal anastomosis using a hybrid or open technique. Stapled anastomosis was performed in most patients. The main histological subtype was adenocarcinoma, and 185 patients received neoadjuvant therapy. All baseline characteristics of the study cohort are shown in the supplementary material, Table S1.

A total of 129 patients, representing 56% of the study cohort, received preoperative PPI therapy. In the univariate analysis, several patient characteristics differed significantly between the two groups including BMI, arterial hypertension, COPD and gastro-esophageal reflux disease. Patients with preoperative PPI therapy showed a slightly increased BMI of 26.3 ± 4.8 kg/m² compared to patients without PPI therapy who had a BMI of 25.5 ± 6.3 kg/m² (*p* = 0.046). Furthermore, 80 of 127 patients with preoperative PPI therapy had arterial hypertension, while only 47 of 102 patients without PPI therapy had arterial hypertension (*p* = 0.032). In addition, patients receiving PPI therapy showed a higher rate of COPD and gastro-esophageal reflux disease compared to those without. The difference in history of gastritis or peptic ulcers did not reach significance between the two groups.

No significant differences were found between the other assessed patient comorbidities, age, ASA status, proportion of neoadjuvant treatment, surgical technique, and postoperative tumor status between the two groups. All patient characteristics are presented in Table 1.

**Table 1:** Patient characteristics

	PPI therapy	No PPI therapy	*P*-value
Gender (% female)	20 (16)	18 (18)	0.721
Age (mean ± SD) in years	65.7 ± 9.4	64.2 ± 9.9	0.241
**BMI (mean ± SD) in kg/m²**	**26.3 ± 4.8**	**25.5 ± 6.3**	**0.046**
Diabetes mellitus Type II (%)	29 (23)	20 (20)	0.746
**Arterial Hypertension (%)**	**80 (62)**	**47 (47)**	**0.032**
**COPD (%)**	**25 (19)**	**9 (9)**	**0.038**
Heart disease (%)	30 (23)	13 (13)	0.060
Vascular disease (%)	26 (20)	15 (15)	0.386
Cardiac arrhythmia (%)	14 (11)	9 (9)	0.825
Chronic kidney disease (%)	5 (4)	3 (3)	1.000
**Gastro-esophageal reflux disease (%)**	**15 (12)**	**3 (3)**	**0.024**
Gastritis or peptic ulcers	16 (12)	6 (6)	0.118
Steroid therapy (%)	8 (6)	4 (4)	0.558
ASA-Grade			0.197
I (%)	1 (1)	1 (1)	
II (%)	21 (19)	24 (25)	
III (%)	86 (78)	69 (72)	
IV (%)	2 (2)	2 (2)	
Neoadjuvant therapy (%)	100 (78)	85 (85)	0.178
T-Status			0.143
0 (%)	37 (29)	26 (26)	
1 (%)	33 (26)	19 (19)	
2 (%)	21 (17)	19 (19)	
3 (%)	36 (28)	36 (36)	
N-Status			1.000
0 (%)	85 (66)	59 (59)	
1 (%)	20 (16)	23 (23)	
2 (%)	13 (10)	9 (9)	
3 (%)	10 (8)	9 (9)	
M-Status			1.000
0 (%)	126 (98)	97 (97)	
1 (%)	3 (2)	3 (3)	
R-Status			1.000
0 (%)	121 (95)	89 (96)	
1(%)	6 (5)	4 (4)	
Histology			0.137
Adenocarcinoma (%)	96 (74)	67 (67)	
Squamous cell carcinoma (%)	30 (24)	33 (33)	
Neuroendocrine tumor (%)	3 (2)	-	
Surgical technique			
Robotic assisted Ivor Lewis esophagectomy (%)	48 (37)	38 (38)	1.000
Open Ivor Lewis esophagectomy (%)	26 (20)	15 (15)	0.386
Hybrid Ivor Lewis esophagectomy (%) (laparoscopy, thoracotomy)	40 (31)	38 (38)	0.325
Minimal invasive Ivor Lewis (%) (laparoscopy, thoracoscopy)	5 (4)	2 (2)	0.473
Mc Keown esophagectomy (%)	10 (7)	7 (7)	1.000
(Open, Hybrid and robotic assisted)			
Anastomosis			0.460
Circular Stapler (%)	117 (91)	94 (94)	
Suture (%)	12 (9)	6 (6)	

*Univariate significant values are displayed in bold

SD = standard deviation

### Anastomotic leak rate and postoperative complications

An anastomotic leak occurred in 20.4% of the entire study cohort. Since three patients were excluded due to early postoperative death, 46 of 226 patients developed anastomotic leak. The univariate comparison showed significantly different anastomotic leak rates between patients with and without preoperative PPI therapy. While an anastomotic leak occurred in 34 patients with preoperative PPI therapy (26.6%), only 12 patients without preoperative PPI therapy (12.2%) developed anastomotic leaks (*p* = 0.008).

**Fig. 2:**
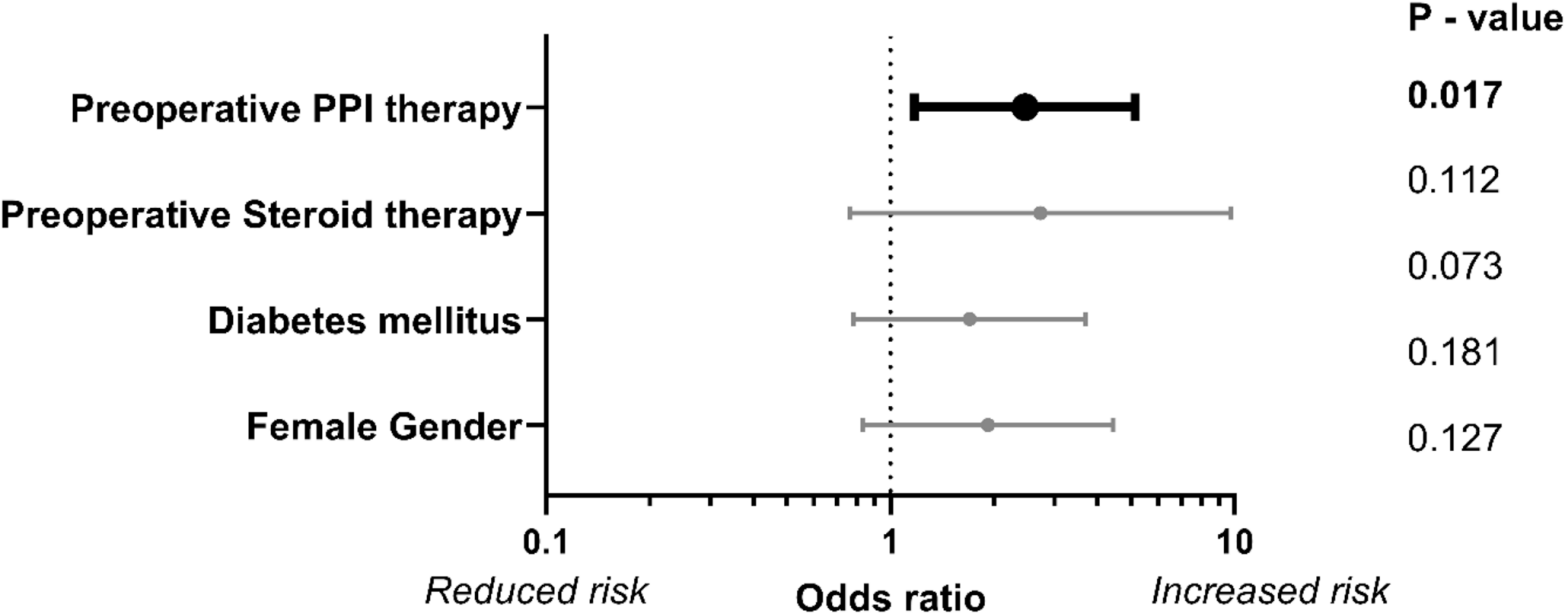
Odds ratios for the risk of anastomotic leak in multivariate, logistic regression. All variables included in the final model are shown

A logistic analysis was performed to estimate variables for the prediction of anastomotic leaks. The final model included gender, diabetes mellitus, preoperative steroid treatment and preoperative PPI therapy. The overall model fit for predicting anastomotic leaks was low (Cox & Snell R² of 0.072 and a Nagelkerke R² of 0.113, *p* = 0.005). Among the selected variables, only preoperative PPI treatment significantly impacted anastomotic leak rate in the multivariate, logistic regression (*p* = 0.017). The calculated odds ratio of 2.5 (95% CI: 1.18–5.15) showed that the patients with preoperative PPI treatment had a 2.5-fold higher risk of anastomotic leak compared to patients without preoperative PPI therapy. The odds ratios of the variables included in logistic regression analyses are presented in Fig. 2 and in Table S2 of the supplemental material.

**Fig. 3:**
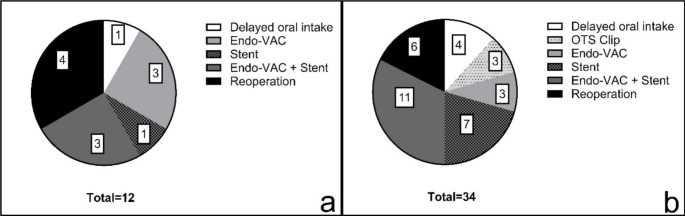
Different therapeutic options for patients with anastomotic leaks, which did not receive preoperative PPI therapy (**a**) and patients with anastomotic leak and preoperative PPI therapy (**b**); Endo-VAC = Endoluminal Vacuum therapy; OTS Clip = Over-the-Scope Clip; Stent = Endoluminal stenting

Patients with preoperative PPI therapy who developed anastomotic leaks tended to have a higher mortality rate (Clavien-Dindo grade V) than patients without PPI therapy. Furthermore, a trend towards a higher rate of single-organ (Clavien-Dindo grade IVa) or multiple-organ (Clavien-Dindo grade IVb) dysfunction was observed in patients with preoperative PPI therapy. Endoscopic procedures were the primary method of treatment for anastomotic leaks in patients with and without preoperative PPI therapy. The Clavien-Dindo grades, which indicate the severity of anastomotic leaks, are listed in Table 2. The various therapeutic options for anastomotic leaks are shown in Fig. 3.

**Table 2:** Postoperative complications of patients with anastomotic leak

	Clavien– Dindo Grade (*n*, %)						
Group	I	II	IIIa	IIIb	IVa	IVb	V
Preoperative PPI therapy	-	-	15 (44)	1 (3)	6 (18)	3 (9)	9 (26)
No preoperative PPI therapy	-	-	5 (42)	2 (17)	1 (8)	2 (17)	2 (17)

The entire study cohort had a postoperative CCI of 31 ± 32.2. Patients with preoperative PPI therapy showed a significantly higher CCI of 34.4 ± 32.9 compared to 26.5 ± 31 of patients without preoperative PPI therapy, *p* = 0.039. Additionally, patients with preoperative PPI therapy had a slightly longer hospital stay (22.8 ± 15 days) compared to patients without preoperative PPI therapy (20.5 ± 18.4 days, *p* = 0.008). However, the multiple, regression analysis did not include preoperative PPI treatment to predict overall, postoperative CCI, while age, cardiac arrhythmia and COPD were found to be significant for predicting postoperative CCI, see Table S3.

## Discussion

For the first time, this retrospective study revealed a higher rate of anastomotic leaks in patients with preoperative PPI therapy compared to patients without preoperative PPI therapy. The multivariate analysis indicated a 2.5-fold increased risk of anastomotic leak in patients with preoperative PPI therapy.

The comparison of patient characteristics revealed a higher prevalence of the comorbidities arterial hypertension, COPD and gastro-esophageal reflux disease and a slightly increased BMI in patients with preoperative PPI therapy. These results may indicate that the less favorable risk profile of patients with preoperative PPI therapy is responsible for the 2.5-fold increased risk of anastomotic leaks. Thus, PPI therapy would only be a confounder of the current study. However, none of the mentioned comorbidities were selected in the multivariate logistic regression analysis to predict anastomotic leaks after esophagectomy. Only preoperative PPI use was found to significantly affect the occurrence of anastomotic leaks. Although a higher rate of overall complications was observed in patients with preoperative PPI therapy compared to those without preoperative PPI therapy, preoperative PPI therapy did not represent a significant risk factor for overall complications in the multivariate analysis of our cohort.

Contrary to expectations, the prevalence of gastro-esophageal reflux disease in the medical records was relatively low. Gastro-esophageal reflux disease is an important factor in the development of Barrett’s mucosa and, thus, a predisposition to esophageal adenocarcinoma. Since 163 patients had an adenocarcinoma in pathological examination, a higher rate of gastro-esophageal reflux disease may be suspected. Consequently, preoperative gastro-esophageal reflux disease itself may be discussed as a risk factor for the occurrence of anastomotic leaks. However, there was no significant difference in the rate of adenocarcinomas in the pathological findings of patients with and without preoperative PPI therapy. Accordingly, a higher rate of gastro-esophageal reflux disease may also be assumed in patients without preoperative PPI therapy.

Furthermore, as a limitation of the retrospective study design, it was not possible to determine the duration of preoperative PPI use or the indication for PPI treatment. Additionally, there may be a significant proportion of patients who frequently take PPI therapy without a prescription or entry in their medical records, since most PPIs are available over the counter [20]. This unknown bias may have affected the present study results.

An already known side effect of PPI therapy, which may be related with to the occurrence of anastomotic leaks, is the development of delayed gastric emptying [11, 12, 21]. For instance, in an early study by Parkman et al. [12], even healthy subjects exhibited delayed gastric emptying in scintigraphy after the administration of omeprazole. As delayed gastric emptying is associated with anastomotic leaks [14, 15], PPIs may increase the anastomotic leak rate by delaying gastric emptying. However, no standardized screening for delayed gastric emptying was performed due to the retrospective design of the current study. Moreover, the development of delayed emptying after esophagectomy is influenced by various surgical [22] and non-surgical factors [23, 24], hindering the establishment of a causal relationship.

Another hypothetical explanation for the increased anastomotic leak rate is a reduced mucosal wound healing under PPI therapy. Particularly, long-term PPI therapy led to distinct changes in mucosal histology, as foveolar epithelial hyperplasia [13] and Enterochromaffin-like cell hyperplasia [10] were described. However, no evidence of reduced mucosal healing after esophagectomy has been found till date. Furthermore, the alteration of the gut microbiome due to PPI therapy [25] might have influenced the anastomotic leak rate. However, the causes for a higher anastomotic leak rate in patients with preoperative PPI therapy cannot be determined by this retrospective study and should be further investigated in future studies.

The presented patient cohort is heterogenous because the surgical techniques varied between January 2016 and November 2024. Different surgical approaches, from open Ivor Lewis or McKeown esophagectomy over hybrid techniques to full robotic approaches, were compared without any subgroup analysis. However, there were no significant differences in the performed surgical procedures between patients with and without preoperative PPI therapy. Accordingly, preoperative PPI therapy proved to be significant for predicting anastomotic leak in these heterogeneous real-world data. Consequently, it may be necessary to discontinue preoperative PPIs for a certain period to reduce the incidence of anastomotic leak. Further prospective randomized studies are needed to confirm our results and to determine the optimal time for preoperative discontinuation of PPI.

Since our study indicates a higher rate of anastomotic leaks in patients with preoperative PPI therapy, postoperative treatment with PPIs should also be critically evaluated. On the one hand, they reduce the rate of anastomotic stenosis in long-term use after esophagectomy. On the other hand, they may lead to anastomotic leaks when used immediately postoperatively, as shown in the present study for preoperative administration. Therefore, prospective, randomized studies should be considered.

## Conclusion

For the first time, a significantly increased anastomotic leak rate after esophagectomy was demonstrated in patients with preoperative PPI therapy compared those without preoperative PPI therapy. A multivariate logistic regression analysis showed a 2.5-fold increased risk of anastomotic leaks. Further prospective studies are necessary to verify the results.

## Electronic supplementary material

Below is the link to the electronic supplementary material.

Supplementary Material 1

Supplementary Material 2

Supplementary Material 3

## Data Availability

Data availability statement: The anonymized patient data is available in the Open Science Framework, URL: https://osf.io/azm9g/?view_only=b45a0afcaefe4576917db0acab1fcfed.

## Declarations

### Previous communication

This research is not based on a previous communication.

## References

[1] Madisch A, Koop H, Miehlke S et al (2023) S2k-Leitlinie gastroösophageale refluxkrankheit und eosinophile ösophagitis der Deutschen gesellschaft für gastroenterologie, Verdauungs- und Stoffwechselkrankheiten (DGVS)– März 2023– AWMF-Registernummer: 021–013. Z Gastroenterol 61:862–933. 10.1055/a-2060-1069 37494073 10.1055/a-2060-1069

[2] Fischbach W, Bornschein J, Hoffmann JC et al (2023) Aktualisierte S2k-Leitlinie Helicobacter pylori und Gastroduodenale ulkuskrankheit der Deutschen gesellschaft für gastroenterologie, Verdauungs- und Stoffwechselkrankheiten (DGVS)– Juli 2022– AWMF-Registernummer: 021–001. Z Gastroenterol 61:544–606. 10.1055/a-1975-0414 37146633 10.1055/a-1975-0414

[3] Alshamsi F, Belley-Cote E, Cook D et al (2016) Efficacy and safety of proton pump inhibitors for stress ulcer prophylaxis in critically ill patients: a systematic review and meta-analysis of randomized trials. Crit Care 120–132. 10.1186/s13054-016-1305-6 10.1186/s13054-016-1305-6 PMC4855320 27142116

[4] Coblijn UK, Lagarde SM, de Castro SMM et al (2016) The influence of prophylactic proton pump inhibitor treatment on the development of symptomatic marginal ulceration in Roux-en-Y gastric bypass patients: a historic cohort study. Surg Obes Relat Dis 12:246–252. 10.1016/j.soard.2015.04.022 26381875 10.1016/j.soard.2015.04.022

[5] Sulieman I, Strobel O, Scharenberg C et al (2020) Symptomatic marginal ulcer after pancreatoduodenectomy. Surgery 168:67–71. 10.1016/j.surg.2020.02.012 32276736 10.1016/j.surg.2020.02.012

[6] Gutschow C, Collard J-M, Romagnoli R et al (2001) Denervated stomach as an esophageal substitute recovers intraluminal acidity with time. Ann Surg 233:509–514 11303132 10.1097/00000658-200104000-00005 PMC1421279

[7] Okuyama M, Motoyama S, Maruyama K et al (2008) Proton pump inhibitors relieve and prevent symptoms related to gastric acidity after esophagectomy. World J Surg 32:246–254. 10.1007/s00268-007-9325-7 18064513 10.1007/s00268-007-9325-7

[8] Yuasa N, Sasaki E, Ikeyama T et al (2005) Acid and duodenogastroesophageal reflux after esophagectomy with gastric tube reconstruction. Official J Am Coll Gastroenterol| ACG 100:1021–1027 10.1111/j.1572-0241.2005.41109.x 15842574

[9] Johansson J, Oberg S, Wenner J et al (2009) Impact of proton pump inhibitors on benign anastomotic stricture formations after esophagectomy and gastric tube reconstruction: results from a randomized clinical trial. Ann Surg 250:667–673. 10.1097/SLA.0b013e3181bcb139 19801933 10.1097/SLA.0b013e3181bcb139

[10] Lundell L, Havu N, Miettinen P et al (2006) Changes of gastric mucosal architecture during long-term Omeprazole therapy: results of a randomized clinical trial. Aliment Pharmacol Ther 23:639–647. 10.1111/j.1365-2036.2006.02792.x 16480403 10.1111/j.1365-2036.2006.02792.x

[11] Rasmussen L, Qvist N, Oster-Jørgensen E et al (1997) A double-blind placebo-controlled study on the effects of Omeprazole on gut hormone secretion and gastric emptying rate. Scand J Gastroenterol 32:900–905. 10.3109/00365529709011199 9299668 10.3109/00365529709011199

[12] Parkman HP, Urbain JL, Knight LC et al (1998) Effect of gastric acid suppressants on human gastric motility. Gut 42:243–250. 10.1136/gut.42.2.243 9536950 10.1136/gut.42.2.243 PMC1726985

[13] Kim GH (2021) Proton pump Inhibitor-Related gastric mucosal changes. Gut Liver 15:646–652. 10.5009/gnl20036 32327613 10.5009/gnl20036 PMC8444106

[14] Sutcliffe RP, Forshaw MJ, Tandon R et al (2008) Anastomotic strictures and delayed gastric emptying after esophagectomy: incidence, risk factors and management. Dis Esophagus 21:712–717. 10.1111/j.1442-2050.2008.00865.x 18847448 10.1111/j.1442-2050.2008.00865.x

[15] Dewar L, Gelfand G, Finley RJ et al (1992) Factors affecting cervical anastomotic leak and stricture formation following esophagogastrectomy and gastric tube interposition. Am J Surg 163:484–489. 10.1016/0002-9610(92)90393-6 1575303 10.1016/0002-9610(92)90393-6

[16] Hagens ERC, Reijntjes MA, Anderegg MCJ et al (2021) Risk factors and consequences of anastomotic leakage after esophagectomy for Cancer. Ann Thorac Surg 112:255–263. 10.1016/j.athoracsur.2020.08.022 33075324 10.1016/j.athoracsur.2020.08.022

[17] Kamarajah SK, Lin A, Tharmaraja T et al (2020) Risk factors and outcomes associated with anastomotic leaks following esophagectomy: a systematic review and meta-analysis. Dis Esophagus 33. 10.1093/dote/doz089 10.1093/dote/doz089 31957798

[18] Low DE, Alderson D, Cecconello I et al (2015) International consensus on standardization of data collection for complications associated with esophagectomy: esophagectomy complications consensus group (ECCG). Ann Surg 262:286–294. 10.1097/SLA.0000000000001098 25607756 10.1097/SLA.0000000000001098

[19] Schindelin J, Arganda-Carreras I, Frise E et al (2012) Fiji: an open-source platform for biological-image analysis. Nat Methods 9:676–682. 10.1038/nmeth.2019 22743772 10.1038/nmeth.2019 PMC3855844

[20] Farrell B, Lass E, Moayyedi P et al (2022) Reduce unnecessary use of proton pump inhibitors. BMJ 379:1–6. 10.1136/bmj-2021-069211 10.1136/bmj-2021-069211 36280250

[21] Sanaka M, Yamamoto T, Kuyama Y (2010) Effects of proton pump inhibitors on gastric emptying: a systematic review. Dig Dis Sci 55:2431–2440. 10.1007/s10620-009-1076-x 20012198 10.1007/s10620-009-1076-x

[22] Akkerman RDL, Haverkamp L, van Hillegersberg R et al (2014) Surgical techniques to prevent delayed gastric emptying after esophagectomy with gastric interposition: a systematic review. Ann Thorac Surg 98:1512–1519. 10.1016/j.athoracsur.2014.06.057 25152385 10.1016/j.athoracsur.2014.06.057

[23] Benedix F, Willems T, Kropf S et al (2017) Risk factors for delayed gastric emptying after esophagectomy. Langenbecks Arch Surg 402:547–554. 10.1007/s00423-017-1576-7 28324171 10.1007/s00423-017-1576-7

[24] Konradsson M, Nilsson M (2019) Delayed emptying of the gastric conduit after esophagectomy. J Thorac Dis 11:835–844. 10.21037/jtd.2018.11.80 10.21037/jtd.2018.11.80 PMC6503290 31080667

[25] Imhann F, Bonder MJ, Vich Vila A et al (2016) Proton pump inhibitors affect the gut Microbiome. Gut 65:740–748. 10.1136/gutjnl-2015-310376 26657899 10.1136/gutjnl-2015-310376 PMC4853569

