# Supplementary material for: Preoperative proton pump inhibitor therapy and anastomotic leak after esophagectomy–a new perspective
Source: Langenbecks Arch Surg. 2025 May 14;410(1):157. doi: 10.1007/s00423-025-03727-3 (PMC12078452; doi:10.1007/s00423-025-03727-3)
Supplement: Supplementary file 1 — Supplementary Material 1 [file 423_2025_3727_MOESM1_ESM.docx]

**Table S1.** Baseline Characteristics

| Variable |  |
| --- | --- |
| PPI therapy (%) | 129 (56) |
| Gender (% female) | 38 (17) |
| Age (mean ± SD) in years | 65 ± 9.6 |
| BMI (mean ± SD) in kg/m² | 26 ± 5.5 |
| Diabetes mellitus (%) | 49 (21) |
| Arterial Hypertension (%) | 127 (55) |
| COPD (%) | 34 (15) |
| Heart disease (%) | 43 (19) |
| Vascular disease (%) | 41 (18) |
| Cardiac arrhythmia (%) | 23 (10) |
| Chronic kidney disease (%) | 8 (4) |
| Gastro-esophageal reflux disease (%) | 18 (8) |
| Gastritis or peptic ulcers (%) | 22 (10) |
| Steroid therapy (%) | 12 (5) |
| ASA-Grade |  |
| I (%) | 1 (1) |
| II (%) | 45 (22) |
| III (%) | 155 (75) |
| IV (%) | 4 (2) |
|  |  |
| Neoadjuvant therapy (%) | 185 (81) |
| T-Status |  |
| 0 (%) | 63 (28) |
| 1 (%) | 52 (23) |
| 2 (%) | 40 (18) |
| 3 (%) | 72 (32) |
|  |  |
| N-Status |  |
| 0 (%) | 144 (63) |
| 1 (%) | 43 (19) |
| 2 (%) | 22 (10) |
| 3 (%) | 19 (8) |
|  |  |
| M-Status |  |
| 0 (%) | 224 (98) |
| 1 (%) | 5 (2) |
|  |  |
| R-Status |  |
| 0 (%) | 210 (96) |
| 1(%) | 10 (4) |
|  |  |
| Histology |  |
| Adenocarcinoma (%) | 163 (71) |
| Squamous cell carcinoma (%) | 63 (28) |
| Neuroendocrine tumor (%) | 2 (1) |
|  |  |
| Surgical technique | |
| Robotic assisted Ivor Lewis esophagectomy (%) | 86 (38) |
| Open Ivor Lewis esophagectomy (%) | 41 (18) |
| Hybrid Ivor Lewis esophagectomy (%)  (laparoscopy, thoracotomy) | 78 (34) |
| Minimal invasive Ivor Lewis esophagectomy (%) | 7 (3) |
| (laparoscopy, thoracoscopy)  McKeown esophagectomy (%)  (Open, Hybrid and robotic assisted) | 17 (7) |
|  |  |
| Anastomosis |  |
| Stapler (%) | 211 (92) |
| Suture (%) | 18 (8) |

SD = standard deviation
