# Supplementary material for: Preoperative proton pump inhibitor therapy and anastomotic leak after esophagectomy–a new perspective
Source: Langenbecks Arch Surg. 2025 May 14;410(1):157. doi: 10.1007/s00423-025-03727-3 (PMC12078452; doi:10.1007/s00423-025-03727-3)
Supplement: Supplementary file 2 — Supplementary Material 2 [file 423_2025_3727_MOESM2_ESM.docx]

**Table S2.** Logistic regression analysis with anastomotic leak as the dependent variable

| Variable | Odds ratio | 95% Confidence interval | P-value |
| --- | --- | --- | --- |
| Diabetes mellitus | 1.70 | 0.78 – 3.69 | 0.181 |
| Female Gender | 1.92 | 0.83 – 4.44 | 0.127 |
| Preoperative PPI therapy | 2.46 | 1.18 – 5.15 | 0.017 |
| Preoperative Steroid therapy | 2.73 | 0.76 – 9.75 | 0.122 |
