# Supplementary material for: Preoperative proton pump inhibitor therapy and anastomotic leak after esophagectomy–a new perspective
Source: Langenbecks Arch Surg. 2025 May 14;410(1):157. doi: 10.1007/s00423-025-03727-3 (PMC12078452; doi:10.1007/s00423-025-03727-3)
Supplement: Supplementary file 3 — Supplementary Material 3 [file 423_2025_3727_MOESM3_ESM.docx]

**Table S3.** Multiple linear regression with the Comprehensive Complication Index (CCI) as dependent variable

| Variable | Coefficient b | Confidence interval | T-value | P-value |
| --- | --- | --- | --- | --- |
| Age | 0.57 | 0.14 – 0.10 | 0.26 | 0.010 |
| Cardiac arrhythmia | -13.88 | - 27.71 – 0.06 | - 2.61 | 0.050 |
| COPD | 15.26 | 3.89 – 26.64 | 2.64 | 0.009 |
